# Effect of Angiogenesis Inhibitor Bevacizumab on Survival in Patients with Cancer: A Meta-Analysis of the Published Literature

**DOI:** 10.1371/journal.pone.0035629

**Published:** 2012-04-23

**Authors:** Yuan Su, Wei-Bing Yang, Shi Li, Zhi-Jian Ye, Huan-Zhong Shi, Qiong Zhou

**Affiliations:** Key Laboratory of Pulmonary Diseases of Health Ministry, Department of Respiratory Diseases, Union Hospital, Tongji Medical College, Huazhong University of Science and Technology, Wuhan, China; Karolinska Institutet, Sweden

## Abstract

Bevacizumab is a recombinant humanized monoclonal antibody against vascular endothelial growth factor which has been used in conjunction with other anti-cancer agents in the treatment of patients with many cancers. It remains controversial whether bevacizumab can prolong survival in cancer patients. This meta-analysis was therefore performed to evaluate effect of bevacizumab on survival in cancer patients. PubMed, EMBASE, and Web of Science databases were searched for English-language studies of randomized controlled trials comparing bevacizumab with control therapy published through February 8, 2012. Progression-free survival, overall survival, and one-year survival rate were analyzed using random- or fixed-effects model. Thirty one assessable randomized controlled trials were identified. A significant improvement in progression-free survival in cancer patients was attributable to bevacizumab compared with control therapy (hazard ratio, 0.72; 95% confidence interval, 0.68 to 0.76; p<0.001). Overall survival was also significantly longer in patients were treated with bevacizumab (hazard ratio, 0.87; 95% confidence interval, 0.83 to 0.91; p<0.001). The significant benefit in one-year survival rate was further seen in cancer patients receiving bevacizumab (odds ratio, 1.30; 95% confidence interval, 1.20 to 1.41; p<0.001). Current evidences showed that bevacizumab prolong progression-free survival and overall survival, and increase one-year survival rate in cancer patients as compared with control therapy.

## Introduction

Angiogenesis is a universal requirement for the growth of solid tumors beyond the limits of oxygen diffusion from the existing vasculature, and plays a crucial role in the growth and metastasis of cancer [1]. Vascular endothelial growth factor (VEGF), a key mediator of angiogenesis, is overexpressed in many tumor types, and has been associated with poor prognosis [1], [2]. The experimental *in vivo* inhibition of the VEGF pathway results in tumor growth inhibition and improves delivery of chemotherapeutic drugs by reducing tumor interstitial fluid pressure and by changing vessel diameter, density, and permeability in response to treatment [3]. These data prompted the clinical investigation of bevacizumab (Avastin; Genentech, South San Francisco, CA), a humanized anti-VEGF monoclonal IgG_1_ antibody in the treatment of cancer patients.

Bevacizumab has shown benefits in the treatment of many types of malignancy including colorectal cancer, non–small cell lung cancer, renal cell carcinoma, breast cancer, and glioblastoma [4]. Bevacizumab monotherapy has been notably less studied in cancer patients than bevacizumab combined with chemotherapy, and fatal adverse events have been reported in cancer patients treated with bevacizumab in combination with chemotherapy [5]. In a recent meta-analysis, Ranpura et al [6] have reported that bevacizumab in combination with chemotherapy or biological therapy was associated with increased treatment-related mortality as compared with chemotherapy alone. To better understand the overall impact of bevacizumab on survival of patients with cancer, we conducted a systematic review and meta-analysis of published randomized controlled trials (RCTs) to evaluate the effect of bevacizumab on progression-free survival (PFS), overall survival (OS), and one-year survival rate (OYSR) in patients with cancer.

## Methods

### Data sources and searches

Two investigators searched PubMed, EMBASE, and Web of Science databases for relevant articles published until February 8, 2012; no lower date limit was applied. We used the following Medical Subject Heading terms and keywords: “bevacizumab”, “Avastin”, and “carcinoma/cancer”, and the searches were limited initially to English publications of RCTs in humans. The search strategy also used text terms such as “progression-free survival”, “overall survival”, “one-year survival rate” and “vascular endothelial growth factor” to identify relevant information. We screened the reference lists of included studies and related publications. The results were then hand searched for eligible trials. Results were double-checked and arbitrated by a second investigator.

### Study selection

We included full-text publications that investigated patients with cancer during treatment with bevacizumab compared with placebo, or bevacizumab-containing chemotherapy regimen with the same regimen either without bevacizumab or with bevacizumab replaced by a placebo, or with different doses of bevacizumab. We excluded studies that were not published as full reports, such as conference abstracts and letters to editors.

### Data extraction and quality assessment

To avoid bias in the data-abstraction process, 2 investigators independently abstracted the data from the trials and subsequently compared the results. The following information was obtained from each report: the first author, the year of publication, the period and location of study, and the numbers of patients enrolled, randomized and analyzed, the proportion of patients who were men, the therapy regimen, the duration of follow up, hazard ratios (HRs) for PFS and OS, and odds ratios (ORs) for OYSR comparing bevacizumab-based therapies with control arms. When studies compared 2 or more doses of bevacizumab with a control, we used data from the group with the highest dose. All data were checked for internal consistency, and disagreements were resolved by discussion among the investigators.

Quality assessment of the publications included was done unblinded by three investigators using a 10 point scoring system as described in a previous meta-analysis [7].

### Statistical analysis

If HRs for PFS or/and OS were not reported in the original publications, we calculated HR values and their 95% confidence intervals (CIs) in each RCT using the abstracted survival probabilities in the Kaplan-Meier curve at specific time points according to the methods proposed by Parmar et al [8]. Minimum and maximum follow-up times were used to estimate censored subjects under the assumption that censoring happens constantly throughout follow-up. If the minimum follow-up time was not available, time zero was substituted for it. HRs were calculated to show how many times higher the probability of death from any cause in patients receiving bevacizumab as compared with those receiving control therapies.

We calculated ORs to assess OYSR advantage of bevacizumab as compared with control therapy. We constructed 2×2 tables from abstracted data for OYSR. ORs and their 95% CIs for the subjects who received bevacizumab relative to those receiving control therapy were calculated from the tables. For OR calculations we excluded ineligible subjects from each evaluation.

A general variance-based method was used to estimate the summary HRs, ORs, and their 95% CIs. We assessed heterogeneity between studies with the *I* ^2^ statistic [9] as a measure of the proportion of total variation in estimates that is due to heterogeneity, where *I* ^2^ value of 50% correspond to cut-off point for a significant heterogeneity. Based on the statistical significance of heterogeneity test, we applied a random-effects model or fixed-effects model to perform meta-analyses. We also used Egger's test [10] to detect possible publication bias.

All statistical analyses were conducted with Comprehensive Meta-Analysis version 2.2.055 software (Englewood, NJ, USA).

## Results

### Eligible RCTs

**Figure 1:**
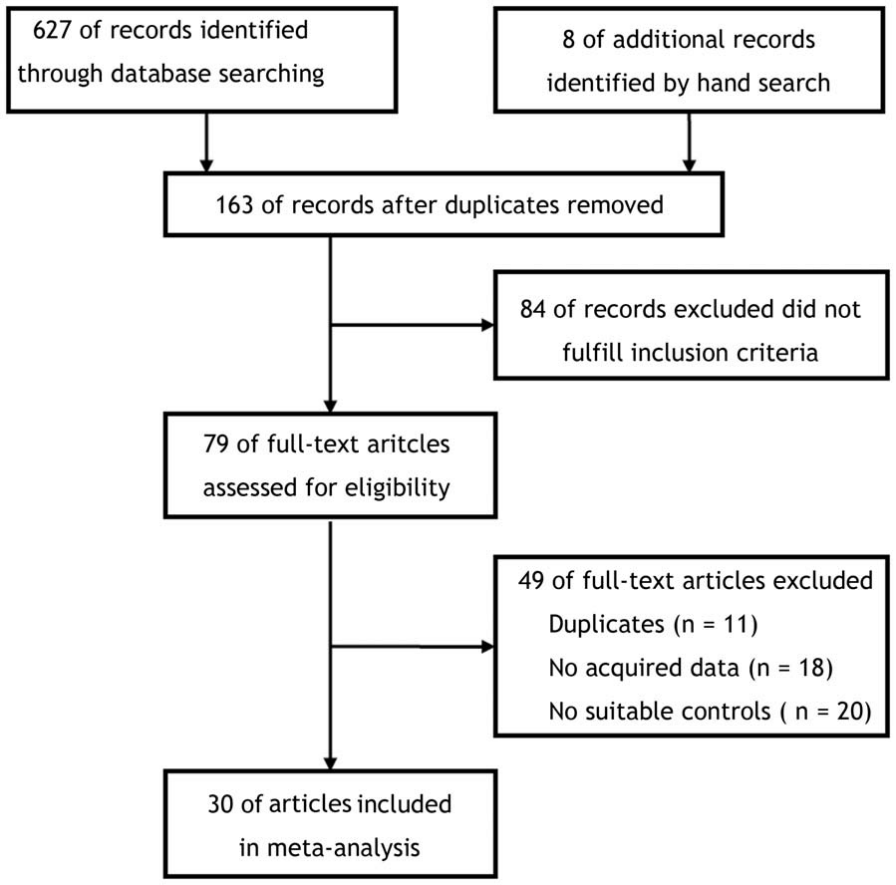
A flow chart showing the progress of trials through the review.

After independent review, seventy-nine publications [11]–[89] reporting RCT results with bevacizumab in patients with various cancers were considered to be eligible for inclusion in the analysis (Figure 1). Of 79 publications, 11 were excluded because the same authors published several reports on the same patients, and only the best-quality study was considered [41]–[51], 18 were excluded because they did not provide acquired data for calculating HR and OR values [52]–[69], 20 were excluded because they did not include suitable control groups [70]–[89]. Subsequently, 30 publications [11]–[40] were available for analyzing the effect of bevacizumab on survival in patients with cancer.

### Study characteristics and quality

Baseline characteristics of the 31 RCTs included in the present meta-analysis are listed in Table S1. These RCTs include 10 phase 2 and 21 phase 3 studies, and they were all published since 2003. Eleven RCTs were from USA, 1 from Germany, 1 from Greece, the remaining 18 from multiple countries (more than 3 countries, including Europe and USA). We noted that the mean of quality scores was 7.6, with a range between 5 and 10 (Table S1). Therefore, the overall quality of all trials was quite good.

### Survival in overall population

**Figure 2:**
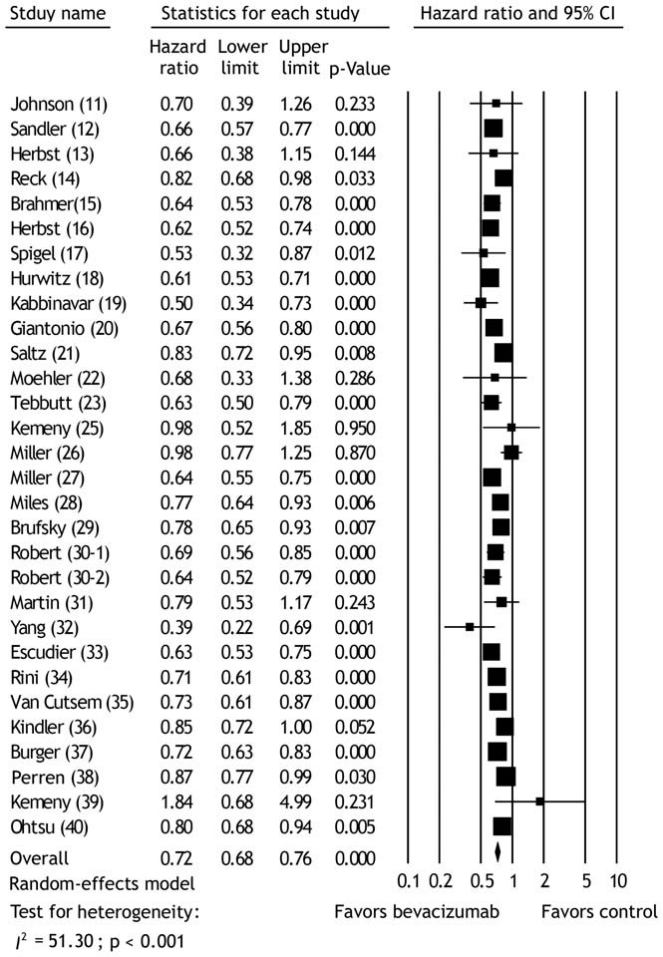
Meta-analysis of the hazard ratios of progression-free survival between bevacizumab and control therapy using random effect model. **Bars, 95% confidence intervals (CI) of hazard ratio in patients receiving bevacizumab versus controls**. The areas of the squares are proportional to the weights used for combining the data. The center of the lozenge gives the combined hazard ratio. The hazard ratio was considered statistically significant if the 95% CI for the overall hazard ratio did not overlap one.

The meta-analysis of PFS was based on 29 publications with 30 RCT s [11]–[23], [25]–[30], [31]–[40], involving 18,132 cancer patients. A statistically significant improvement in PFS was observed favoring bevacizumab groups compared with control groups (pooled HR, 0.72; 95% CI, 0.68 to 0.76; p<0.001; random-effects model) (Figure 2). Overall test for heterogeneity showed that *I* ^2^ = 51.30 (p<0.001), indicating a significant heterogeneity between studies. Evaluation of publication bias showed that the Egger test was not significant (p = 0.669). The funnel plots for publication bias also showed an apparent symmetry (data not shown). These results indicated that there was no publication bias.

**Figure 3:**
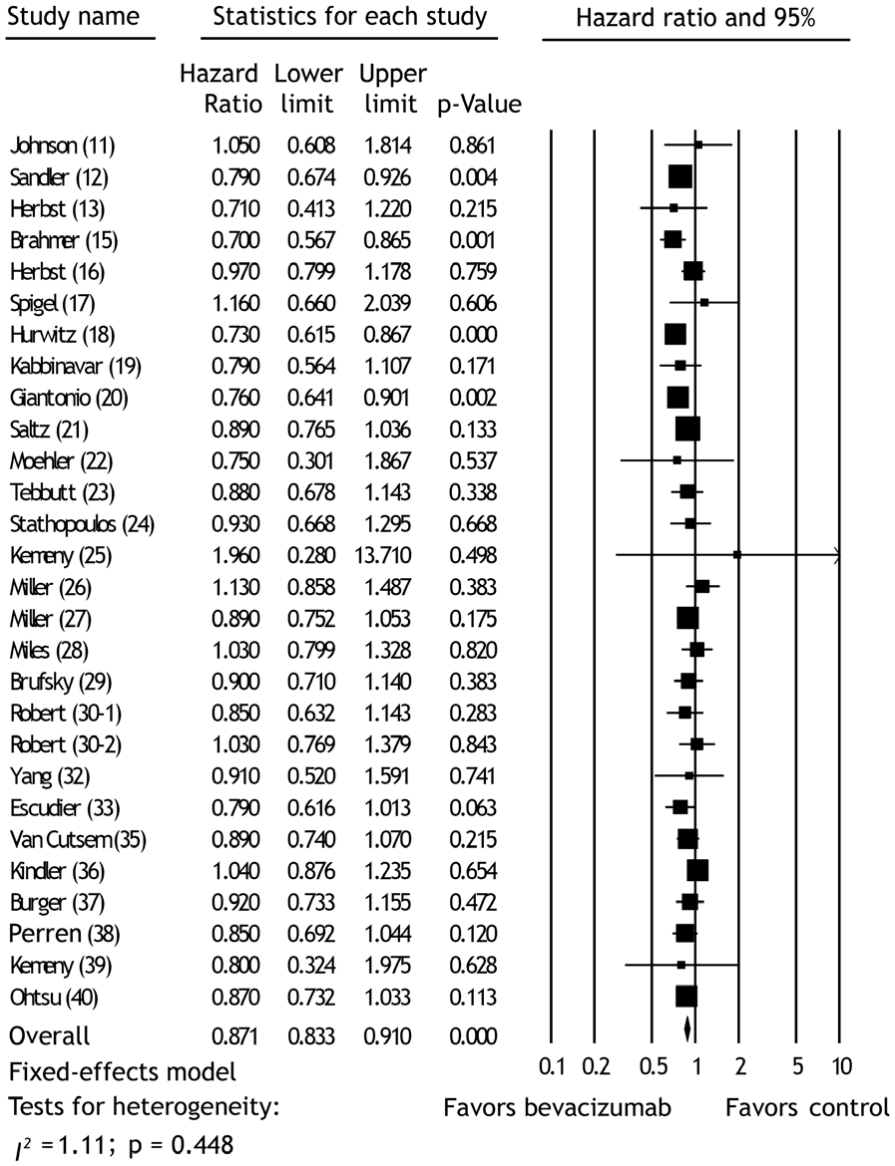
Meta-analysis of the hazard ratios of overall survival between bevacizumab and control therapy using random effect model. Bars, 95% confidence intervals (CI) of hazard ratio in patients receiving bevacizumab versus controls. The areas of the squares are proportional to the weights used for combining the data. The center of the lozenge gives the combined hazard ratio. The hazard ratio was considered statistically significant if the 95% CI for the overall hazard ratio did not overlap one.

The meta-analysis of OS was based on 27 publications with 28 RCTs [11]–[13], [15]–[30], [32]–[33], [35]–[40], involving 16,462 cancer patients. Bevacizumab had improvement in OS as compared with control therapy (HR, 0.87; 95% CI, 0.83 to 0.91; p<0.001; fixed-effects model) (Figure 3). Overall test for heterogeneity showed that *I* ^2^ = 1.11 (p = 0.448), indicating no heterogeneity between studies. We recorded no evidence of publication bias with the Egger test (p = 0.540).

**Figure 4:**
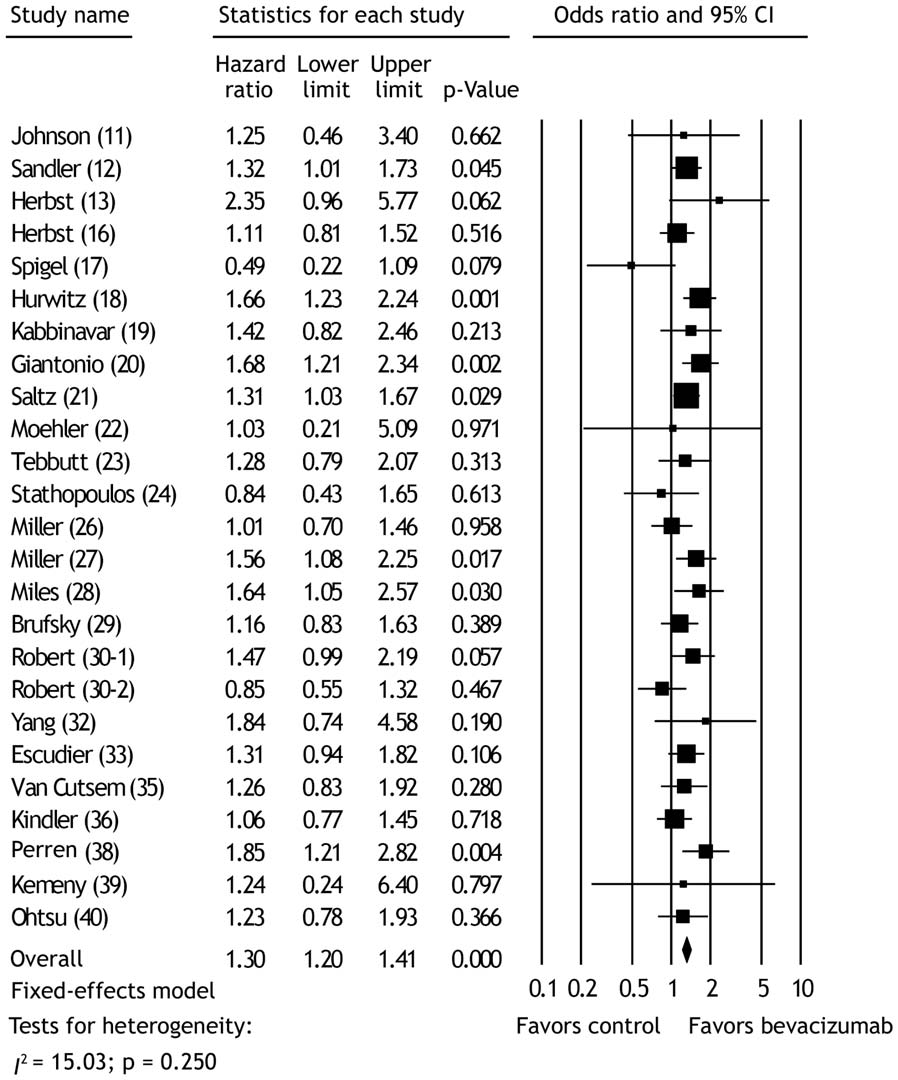
Meta-analysis of the odds ratios of one-year survival rate between bevacizumab and control therapy. Bars, 95% confidence intervals (CI) of odds ratio in patients receiving bevacizumab versus controls. The areas of the squares are proportional to the weights used for combining the data. The center of the lozenge gives the combined odds ratio. The odds ratio was considered statistically significant if the 95% CI for the overall odds ratio did not overlap one.

The OR values of OYSR for meta-analysis were available or have been computed from 24 publications with 25 RCTs [11]–[13], [16]–[24], [26]–[30], [32], [33], [35], [36], [38]–[40]. The analysis showed significant improvement in OYSR for bevacizumab versus control (OR = 1.30; 95% CI, 1.20 to 1.41; p<0.001; fixed-effects model) (Figure 4). Overall test for heterogeneity showed that *I* ^2^ = 15.03 (p = 0.250), indicating no heterogeneity between studies. We recorded no evidence of publication bias with the Egger test (p = 0.559).

Bevacizumab monotherapy was administered in only one RCT [32], in the remaining 30 RCTs, bevacizumab was combined with chemotherapy. After excluding the RCT with bevacizumab monotherapy, HRs of PFS and OS with bevacizumab remained similar and was 0.72 (95% CI, 0.67 to 0.76; p<0.001) and 0.87 (95% CI, 0.83 to 0.91; p<0.001), respectively; OR of OYSR also remained similar and was 1.30 (95% CI, 1.20 to 1.42; p<0.001).

### Subgroup analyses

Based on the statistical significance of heterogeneity test, a random-effects model or fixed-effects model was used to alalyze the effects of bevacizumab on survival in patients with cancers in following subgroups.

From 7 RCTs with lung cancer [11]–[17], 7 with colorectal cancer [18]–[23], [25], 7 with breast cancer [26]–[30], [31], 3 with renal cell carcinoma [32]–[34], 2 with pancreatic cancer [35], [36], 2 with ovarian cancer [37], [38], and 2 with the other cancers (liver cancer and gastric cancer) [39], [40], data could be obtained for analyzing PFS. Our data showed that bevacizumab was associated with significant improvement in PFS in patients with all kinds of cancers, except for liver cancer and gastric cancer (Table S2). From 6 RCTs with lung cancer [11]–[13], [15]–[17], 8 with colorectal cancer [18]–[25], 6 with breast cancer [26]–[30(1, 2)], 2 with renal cell carcinoma [32]–[33], and 2 with pancreatic cancer [35], [36], 2 with ovarian cancer [37]–[38],1 liver cancer and 1 gastric cancer [39], [40], data could be obtained for analyzing OS. From 5 RCTs with lung cancer [11]–[13], [16]–[17], 7 with colorectal cancer [18]–[24], 6 with breast cancer [26]–[30(1, 2)], 2 with renal cell carcinoma [32]–[33], and 2 with pancreatic cancer [35], [36], 1 with ovarian cancer [38], 1 liver cancer and 1 gastric cancer [39], [40], data could be obtained for analyzing OYSR. Also as shown in Table S2, bevacizumab improved OS in patients with lung cancer and colorectal cancer, but not with renal cell carcinoma, breast cancer, pancreatic cancer, ovarian cancer, liver cancer and gastric cancer. It was found that bevacizumab had benefit in improvement of OYSR in patients with colorectal cancer, breast cancer and ovarian cancer, but not with lung cancer, renal cell carcinoma, pancreatic cancer, liver cancer and gastric cancer.

From 22 RCTs [11]–[17], [20], [22], [26]–[30], [31]–[34], [36]–[37], [39] of bevacizumab at an equivalent of 5.0 mg/kg per week or more (high dose) and 12 RCTs [11], [14], [18]–[19], [21], [23], [25], [28], [32], [35], [38], [40] of bevacizumab at 2.5 mg/kg per week (low dose), data could be obtained for analyzing PFS. From 19 RCTs [11]–[13], [15]–[17], [20], [22], [26]–[30], [32]–[33], [36]–[37], [39] of high-dose bevacizumab and 12 RCTs [11], [18]–[19], [21], [23]–[25], [28], [32], [35], [38], [40] of low dose bevacizumab, data could be obtained for analyzing OS. From 17 RCTs [11]–[13], [16]–[17], [20], [22], [26]–[30], [32]–[33], [36], [39] of high-dose bevacizumab and 11 RCTs [11], [18]–[19], [21], [23]–[24], [28], [32], [35], [38], [40] of low dose bevacizumab, data could be obtained for analyzing OYSR. Our analysis revealed that the cancer patients treated with both high and low doses of bevacizumab showed better PFS and OS benefits compared with those treated with control therapies (Table S2). Similar to PFS and OS results, the cancer patients treated with both high and low doses of bevacizumab also showed a better benefit on OYSR compared with those treated with control therapies (Table S2). Overall, no statistically significant difference was found for the effect of bevacizumab on PFS, OS and OYSR between the high and low doses of bevacizumab (all p>0.05).

To determine whether the type of chemotherapeutic agent may alter the impact of bevacizumab on patients' survival, we performed a subgroup analysis stratified according to drug class such as platinum (cisplatin, carboplatin, or oxaliplatin) and taxanes (paclitaxel or docetaxel) [11]–[15], [17], [20]–[21], [27]–[30], [31], [37]–[38], [40] versus others (nonplatinum- and nontaxane-based chemotherapies including fluorouracil, irinotecan, and gemcitabine) [16], [18]–[19], [22]–[26], [30], [33]–[36], [39]. Also shown in Table S2, the HR values of PFS and OS, and the OR value of OYSR for bevacizumab with platinum- or taxane-containing regimens were similar to those for nonplatinum- or nontaxane-based regimens. This difference in risk of PFS, OS and OYSR with bevacizumab among these chemotherapeutic classes was not statistically significant (all p>0.05).

## Discussion

Most cancers are diagnosed with unresectable advanced disease [90]. Systemic chemotherapy or radiotherapy is indicated for the cancer patients with advanced disease to prolong survival, control symptoms and maintain or improve quality of life. However, the benefit of chemoradiotherapy is counterbalanced by increased and prohibitive toxicity, particularly among cancer patients with coexisting medical conditions and decreased performance status. Therefore, novel therapeutic strategies are needed. Bevacizumab can bind selectively circulating VEGF, and thus inhibits the binding of VEGF to its cell surface receptors. This inhibition leads to a reduction in microvascular growth of tumor blood vessels and thus limits the blood supply to tumor tissues [91]. As a matter of fact, bevacizumab has been used in conjunction with other anti-cancer agents in the treatment of patients with many cancers.

Recently, several meta-analyses revealed that the use of bevacizumab was associated with increased risks of arterial thromboembolism [92], venous thromboembolism [93], gastrointestinal perforation [94], severe proteinuria [95], and high-grade hypertension [96], and treatment-related mortality [6]. Although the inhibition of VEGF by bevacizumab has been noted to cause serious adverse events, evidence has continued to accumulate that bevacizumab is a powerful anti-angiogenic agent that has efficacy in the treatment of a wide variety of cancers. The present meta-analysis has shown the benefit of bevacizumab in the treatment of patients with cancer. A significant improvement in PFS, OS and OYSR was seen in overall population with cancer receiving bevacizumab-based therapies when compared with control therapies.

We failed to perform the meta-regression analysis to assess the effect of bevacizumab monotherapy or combination with chemotherapy on cancer patients' survival, since bevacizumab monotherapy was studied in only one RCT [32]. After excluding the RCT with bevacizumab monotherapy, HRs of PFS and OS with bevacizumab remained similar and were all significant improved. Therefore, according to the results of the present meta-analysis, the addition of bevacizumab to first-line chemotherapy regimens would provide a significant advantage in terms of PFS, OS, and OYSR.

We evaluated impact of bevacizumab on cancer patients' survival according to tumor type, and noted that bevacizumab improved in PFS in patients with most of cancers studied except for liver cancer and gastric cancer, but did not improve OS in patients with renal cell carcinoma, breast cancer, pancreatic cancer, ovarian cancer, liver cancer and gastric cancer, did not improved OYSR in patients with lung cancer, renal cell carcinoma pancreatic cancer, ovarian cancer, liver cancer and gastric cancer. These data suggested that patients with some kinds of cancers, such as lung cancer and colorectal cancer, would obtain more survival benefit from bevacizumab therapy compared with the other kinds.

Our subgroup analysis revealed that the cancer patients treated with both high and low doses of bevacizumab showed better PFS, OS, and OYSR benefits compared with those treated with control therapies. Overall, no statistically significant difference was found for the effect of bevacizumab on PFS, OS and OYSR between the high and low doses of bevacizumab. These data suggested that cancer patients treated with higher dose of bevacizumab did not have more favorable benefit in PFS, OS, as well as OYSR than those treated with lower dose. Since lower dose were as effective as higher doses, and higher dose is associated with significantly increased risk with serious adverse events [6], and thus should be the recommended for patients with cancer in case of need.

Although the primary aim of the present meta-analysis was to to evaluate effect of bevacizumab on survival in cancer patients, one should also pay attention to the adverse events risk associated with bevacizumab. Recent meta-analyses have shown that bevacizumab could increase the risk of left ventricular dysfunction and hemorrhagic events, and even were associated with fatal adverse events, including treatment-related mortality [6], [97]. The interaction between bevacizumab and certain chemotherapeutic agents might also affect the effects of bevacizumab on cancer patients' survival. However, our further subgroup analysis showed HR values of PFS and OS, and the OR values of OYSR for bevacizumab with platinum- or taxane-containing regimens were similar to those for nonplatinum- or nontaxane-based regimens. The difference in risk of PFS, OS and OYSR with bevacizumab among these chemotherapeutic classes was not statistically significant.

Several technical issues have to be mentioned in relation to this meta-analysis. This meta-analysis was not based on individual patient data and was not subjected to an open external evaluation procedure. Meta-analyses based on published data tend to overestimate treatment effects compared with individual patient data analyses. However, analyses using individual patient data may include fewer studies if all authors do not agree to submit their full databases to the analyzing group. Another drawback of analyses based on individual patient data is the time-consuming review process. The results must therefore be interpreted cautiously, as an individual patient data-based meta-analysis would give more reliable estimation than one based on abstracted data [98]. Publication bias is a significant threat to the validity of the results, however, such a situation did not exist in the present meta-analysis. Heterogeneity among trials may be another limitation of our meta-analysis, even though we applied a random-effects model that takes possible heterogeneity into consideration. The accuracy of the values of HR and OR estimated from the Kaplan-Meier curves is another important issue. We obtained fairly good correlation between the HRs and ORs reported in this article and those obtained based on the Kaplan-Meier curves, suggesting that curve-based HRs or/and ORs can be substituted in cases where the HRs or/and ORs are not available.

In conclusion, our results have demonstrated that PFS, OS, as well as OYSR was significantly improved in cancer patients treated with bevacizumab as compared with control therapies.

## Supporting Information

Table S1 **Characteristics of the trials included in this meta-analysis.**
(DOC)

Table S2 **Subgroup analyses.**
(DOC)

## References

[1] Kerbel RS (2008). Tumor angiogenesis.. N Engl J Med.

[2] Hicklin DJ, Ellis LM (2005). Role of the vascular endothelial growth factor pathway in tumor growth and angiogenesis.. J Clin Oncol.

[3] Gerber HP, Ferrara N (2005). Pharmacology and pharmacodynamics of bevacizumab as monotherapy or in combination with cytotoxic therapy in preclinical studies.. Cancer Res.

[4] Van Meter ME, Kim ES (2010). Bevacizumab: current updates in treatment.. Curr Opin Oncol.

[5] Gressett SM, Shah SR (2009). Intricacies of bevacizumab-induced toxicities and their management.. Ann Pharmacother.

[6] Ranpura V, Hapani S, Wu S (2011). Treatment-related mortality with bevacizumab in cancer patients: a meta-analysis.. JAMA.

[7] Jiang J, Shi HZ, Deng JM, Liang QL, Qin SM (2009). Efficacy of intensified chemotherapy with hematopoietic progenitors in small-cell lung cancer: a meta-analysis of the published literature.. Lung Cancer.

[8] Parmar MK, Torri V, Stewart L (1998). Extracting summary statistics to perform meta-analyses of the published literature for survival endpoints.. Stat Med.

[9] Higgins JP, Thompson SG (2002). Quantifying heterogeneity in a meta-analysis.. Stat Med.

[10] Egger M, Davey Smith G, Schneider M, Minder C (1997). Bias in meta-analysis detected by a simple, graphical test.. BMJ.

[11] Johnson DH, Fehrenbacher L, Novotny WF, Herbst RS, Nemunaitis JJ (2004). Randomized phase II trial comparing bevacizumab plus carboplatin and paclitaxel with carboplatin and paclitaxel alone in previously untreated locally advanced or metastatic non-small-cell lung cancer.. J Clin Oncol.

[12] Sandler A, Gray R, Perry MC, Brahmer J, Schiller JH (2006). Paclitaxel-carboplatin alone or with bevacizumab for non-small-cell lung cancer.. N Engl J Med.

[13] Herbst RS, O'Neill VJ, Fehrenbacher L, Belani CP, Bonomi PD (2007). Phase II study of efficacy and safety of bevacizumab in combination with chemotherapy or erlotinib compared with chemotherapy alone for treatment of recurrent or refractory non small-cell lung cancer.. J Clin Oncol.

[14] Reck M, von Pawel J, Zatloukal P, Ramlau R, Gorbounova V (2009). Phase III trial of cisplatin plus gemcitabine with either placebo or bevacizumab as first-line therapy for nonsquamous non-small-cell lung cancer: AVAil.. J Clin Oncol.

[15] Brahmer JR, Dahlberg SE, Gray RJ, Schiller JH, Perry MC (2011). Sex differences in outcome with bevacizumab therapy: analysis of patients with advanced-stage non-small cell lung cancer treated with or without bevacizumab in combination with paclitaxel and carboplatin in the Eastern Cooperative Oncology Group Trial 4599.. J Thorac Oncol.

[16] Herbst RS, Ansari R, Bustin F, Flynn P, Hart L (2011). Efficacy of bevacizumab plus erlotinib versus erlotinib alone in advanced non-small-cell lung cancer after failure of standard first-line chemotherapy (BeTa): a double-blind, placebo-controlled, phase 3 trial.. Lancet.

[17] Spigel DR, Townley PM, Waterhouse DM, Fang L, Adiguzel I (2011). Randomized phase II study of bevacizumab in combination with chemotherapy in previously untreated extensive-stage small-cell lung cancer: results from the SALUTE trial.. J Clin Oncol.

[18] Hurwitz H, Fehrenbacher L, Novotny W, Cartwright T, Hainsworth J (2004). Bevacizumab plus irinotecan, fluorouracil, and leucovorin for metastatic colorectal cancer.. N Engl J Med.

[19] Kabbinavar FF, Schulz J, McCleod M, Patel T, Hamm JT (2005). Addition of bevacizumab to bolus fluorouracil and leucovorin in first-line metastatic colorectal cancer: results of a randomized phase II trial.. J Clin Oncol.

[20] Giantonio BJ, Catalano PJ, Meropol NJ, O'Dwyer PJ, Mitchell EP (2007). Bevacizumab in combination with oxaliplatin, fluorouracil, and leucovorin (FOLFOX4) for previously treated metastatic colorectal cancer: results from the Eastern Cooperative Oncology Group Study E3200.. J Clin Oncol.

[21] Saltz LB, Clarke S, Díaz-Rubio E, Scheithauer W, Figer A (2008). Bevacizumab in combination with oxaliplatin-based chemotherapy as first-line therapy in metastatic colorectal cancer: a randomized phase III study.. J Clin Oncol.

[22] Moehler M, Sprinzl MF, Abdelfattah M, Schimanski CC, Adami B (2009). Capecitabine and irinotecan with and without bevacizumab for advanced colorectal cancer patients.. World J Gastroenterol.

[23] Tebbutt NC, Wilson K, Gebski VJ, Cummins MM, Zannino D (2010). Capecitabine, bevacizumab, and mitomycin in first-line treatment of metastatic colorectal cancer: results of the Australasian Gastrointestinal Trials Group Randomized Phase III MAX Study.. J Clin Oncol.

[24] Stathopoulos GP, Batziou C, Trafalis D, Koutantos J, Batzios S (2010). Treatment of Colorectal Cancer with and without Bevacizumab: A Phase III Study.. Oncology.

[25] Kemeny NE, Jarnagin WR, Capanu M, Fong Y, Gewirtz AN (2011). Randomized phase II trial of adjuvant hepatic arterial infusion and systemic chemotherapy with or without bevacizumab in patients with resected hepatic metastases from colorectal cancer.. J Clin Oncol.

[26] Miller KD, Chap LI, Holmes FA, Cobleigh MA, Marcom PK (2005). Randomized phase III trial of capecitabine compared with bevacizumab plus capecitabine in patients with previously treated metastatic breast cancer.. J Clin Oncol.

[27] Miller K, Wang M, Gralow J, Dickler M, Cobleigh M (2007). Paclitaxel plus bevacizumab versus paclitaxel alone for metastatic breast cancer.. N Engl J Med.

[28] Miles DW, Chan A, Dirix LY, Cortés J, Pivot X (2010). Phase III study of bevacizumab plus docetaxel compared with placebo plus docetaxel for the first-line treatment of human epidermal growth factor receptor 2-negative metastatic breast cancer.. J Clin Oncol.

[29] Brufsky AM, Hurvitz S, Perez E, Swamy R, Valero V (2011). RIBBON-2: a randomized, double-blind, placebo-controlled, phase III trial evaluating the efficacy and safety of bevacizumab in combination with chemotherapy for second-line treatment of human epidermal growth factor receptor 2-negative metastatic breast cancer.. J Clin Oncol.

[30] Robert NJ, Diéras V, Glaspy J, Brufsky AM, Bondarenko I (2011). RIBBON-1: randomized, double-blind, placebo-controlled, phase III trial of chemotherapy with or without bevacizumab for first-line treatment of human epidermal growth factor receptor 2-negative, locally recurrent or metastatic breast cancer.. J Clin Oncol.

[31] Martin M, Roche H, Pinter T, Crown J, Kennedy MJ (2011). Motesanib, or open-label bevacizumab, in combination with paclitaxel, as first-line treatment for HER2-negative locally recurrent or metastatic breast cancer: a phase 2, randomised, double-blind, placebo-controlled study.. Lancet Oncol.

[32] Yang JC, Haworth L, Sherry RM, Hwu P, Schwartzentruber DJ (2003). A randomized trial of bevacizumab, an anti-vascular endothelial growth factor antibody, for metastatic renal cancer.. N Engl J Med.

[33] Escudier B, Pluzanska A, Koralewski P, Ravaud A, Bracarda S (2007). Bevacizumab plus interferon alfa-2a for treatment of metastatic renal cell carcinoma: a randomised, double-blind phase III trial.. Lancet.

[34] Rini BI, Halabi S, Rosenberg JE, Stadler WM, Vaena DA (2008). Bevacizumab plus interferon alfa compared with interferon alfa monotherapy in patients with metastatic renal cell carcinoma: CALGB 90206.. J Clin Oncol.

[35] Van Cutsem E, Vervenne WL, Bennouna J, Humblet Y, Gill S (2009). Phase III trial of bevacizumab in combination with gemcitabine and erlotinib in patients with metastatic pancreatic cancer.. J Clin Oncol.

[36] Kindler HL, Niedzwiecki D, Hollis D, Sutherland S, Schrag D (2010). Gemcitabine plus bevacizumab compared with gemcitabine plus placebo in patients with advanced pancreatic cancer: phase III trial of the Cancer and Leukemia Group B (CALGB 80303).. J Clin Oncol.

[37] Burger RA, Brady MF, Bookman MA, Fleming GF, Monk BJ (2011). Incorporation of bevacizumab in the primary treatment of ovarian cancer.. N Engl J Med.

[38] Perren TJ, Swart AM, Pfisterer J, Ledermann JA, Pujade-Lauraine E (2011). A Phase 3 Trial of Bevacizumab in Ovarian Cancer.. N Engl J Med.

[39] Kemeny NE, Schwartz L, Gönen M, Yopp A, Gultekin D (2011). Treating primary liver cancer with hepatic arterial infusion of floxuridine and dexamethasone: does the addition of systemic bevacizumab improve results?. Oncology.

[40] Ohtsu A, Shah MA, Van Cutsem E, Rha SY, Sawaki A (2011). Bevacizumab in combination with chemotherapy as first-line therapy in advanced gastric cancer: a randomized, double-blind, placebo-controlled phase III study.. J Clin Oncol.

[41] Jubb AM, Hurwitz HI, Bai W, Holmgren EB, Tobin P (2006). Impact of vascular endothelial growth factor-A expression, thrombospondin-2 expression, and microvessel density on the treatment effect of bevacizumab in metastatic colorectal cancer.. J Clin Oncol.

[42] Kabbinavar FF, Wallace JF, Holmgren E, Yi J, Cella D (2008). Health-related quality of life impact of bevacizumab when combined with irinotecan, 5-fluorouracil, and leucovorin or 5-fluorouracil and leucovorin for metastatic colorectal cancer.. Oncologist.

[43] Melichar B, Koralewski P, Ravaud A, Pluzanska A, Bracarda S (2008). First-line bevacizumab combined with reduced dose interferon-alpha2a is active in patients with metastatic renal cell carcinoma.. Ann Oncol.

[44] Ramalingam SS, Dahlberg SE, Langer CJ, Gray R, Belani CP (2008). Outcomes for elderly, advanced-stage non small-cell lung cancer patients treated with bevacizumab in combination with carboplatin and paclitaxel: analysis of Eastern Cooperative Oncology Group Trial 4599.. J Clin Oncol.

[45] Gray R, Bhattacharya S, Bowden C, Miller K, Comis RL (2009). Independent review of E2100: a phase III trial of bevacizumab plus paclitaxel versus paclitaxel in women with metastatic breast cancer.. J Clin Oncol.

[46] Hurwitz HI, Yi J, Ince W, Novotny WF, Rosen O (2009). The clinical benefit of bevacizumab in metastatic colorectal cancer is independent of K-ras mutation status: analysis of a phase III study of bevacizumab with chemotherapy in previously untreated metastatic colorectal cancer.. Oncologist.

[47] Okines A, Puerto OD, Cunningham D, Chau I, Van Cutsem E (2009). Surgery with curative-intent in patients treated with first-line chemotherapy plus bevacizumab for metastatic colorectal cancer First BEAT and the randomized phase-III NO16966 trial.. Br J Cancer.

[48] Escudier B, Bellmunt J, Négrier S, Bajetta E, Melichar B (2010). Phase III trial of bevacizumab plus interferon alfa-2a in patients with metastatic renal cell carcinoma (AVOREN): final analysis of overall survival.. J Clin Oncol.

[49] Rini BI, Halabi S, Rosenberg JE, Stadler WM, Vaena DA (2010). Phase III trial of bevacizumab plus interferon alfa versus interferon alfa monotherapy in patients with metastatic renal cell carcinoma: final results of CALGB 90206.. J Clin Oncol.

[50] Mok TS, Hsia TC, Tsai CM, Tsang K, Chang GC (2011). Efficacy of bevacizumab with cisplatin and gemcitabine in Asian patients with advanced or recurrent non-squamous non-small cell lung cancer who have not received prior chemotherapy: a substudy of the Avastin in Lung trial.. Asia Pac J Clin Oncol.

[51] Price TJ, Hardingham JE, Lee CK, Weickhardt A, Townsend AR (2011). Impact of KRAS and BRAF Gene Mutation Status on Outcomes From the Phase III AGITG MAX Trial of Capecitabine Alone or in Combination With Bevacizumab and Mitomycin in Advanced Colorectal Cancer.. J Clin Oncol.

[52] Kabbinavar F, Hurwitz HI, Fehrenbacher L, Meropol NJ, Novotny WF (2003). Phase II, randomized trial comparing bevacizumab plus fluorouracil (FU)/leucovorin (LV) with FU/LV alone in patients with metastatic colorectal cancer.. J Clin Oncol.

[53] Price N (2004). Bevacizumab improves the efficacy of 5-fluorouracil/leucovorin in patients with advanced colorectal cancer.. Clin Colorectal Cancer.

[54] Rini BI, Halabi S, Taylor J, Small EJ, Schilsky RL (2004). Cancer and Leukemia Group B 90206: A randomized phase III trial of interferon-alpha or interferon-alpha plus anti-vascular endothelial growth factor antibody (bevacizumab) in metastatic renal cell carcinoma.. Clin Cancer Res.

[55] Hurwitz HI, Fehrenbacher L, Hainsworth JD, Heim W, Berlin J (2005). Bevacizumab in combination with fluorouracil and leucovorin: an active regimen for first-line metastatic colorectal cancer.. J Clin Oncol.

[56] Kabbinavar FF, Hambleton J, Mass RD, Hurwitz HI, Bergsland E (2005). Combined analysis of efficacy: the addition of bevacizumab to fluorouracil/leucovorin improves survival for patients with metastatic colorectal cancer.. J Clin Oncol.

[57] Hochster HS, Hart LL, Ramanathan RK, Childs BH, Hainsworth JD (2008). Safety and efficacy of oxaliplatin and fluoropyrimidine regimens with or without bevacizumab as first-line treatment of metastatic colorectal cancer: results of the TREE Study.. J Clin Oncol.

[58] Stein WD, Yang J, Bates SE, Fojo T (2008). Bevacizumab reduces the growth rate constants of renal carcinomas: a novel algorithm suggests early discontinuation of bevacizumab resulted in a lack of survival advantage.. Oncologist.

[59] Allegra CJ, Yothers G, O'Connell MJ, Sharif S, Colangelo LH (2009). Initial safety report of NSABP C-08: A randomized phase III study of modified FOLFOX6 with or without bevacizumab for the adjuvant treatment of patients with stage II or III colon cancer.. J Clin Oncol.

[60] Robertson JD, Botwood NA, Rothenberg ML, Schmoll HJ (2009). Phase III trial of FOLFOX plus bevacizumab or cediranib (AZD2171) as first-line treatment of patients with metastatic colorectal cancer: HORIZON III.. Clin Colorectal Cancer.

[61] Ning YM, Gulley JL, Arlen PM, Woo S, Steinberg SM (2010). Phase II trial of bevacizumab, thalidomide, docetaxel, and prednisone in patients with metastatic castration-resistant prostate cancer.. J Clin Oncol.

[62] Pivot X, Schneeweiss A, Verma S, Thomssen C, Passos-Coelho JL (2011). Efficacy and safety of bevacizumab in combination with docetaxel for the first-line treatment of elderly patients with locally recurrent or metastatic breast cancer: results from AVADO.. Eur J Cancer.

[63] Salama JK, Haraf DJ, Stenson KM, Blair EA, Witt ME (2011). A randomized phase II study of 5-fluorouracil, hydroxyurea, and twice-daily radiotherapy compared with bevacizumab plus 5-fluorouracil, hydroxyurea, and twice-daily radiotherapy for intermediate-stage and T4N0-1 head and neck cancers.. Ann Oncol.

[64] Soria JC, Márk Z, Zatloukal P, Szima B, Albert I (2011). Randomized phase II study of dulanermin in combination with paclitaxel, carboplatin, and bevacizumab in advanced non-small-cell lung cancer.. J Clin Oncol.

[65] Reardon DA, Desjardins A, Peters KB, Vredenburgh JJ, Gururangan S (2011). Phase 2 study of carboplatin, irinotecan, and bevacizumab for recurrent glioblastoma after progression on bevacizumab therapy.. Cancer.

[66] Reardon DA, Desjardins A, Peters K, Gururangan S, Sampson J (2011). Phase II study of metronomic chemotherapy with bevacizumab for recurrent glioblastoma after progression on bevacizumab therapy.. J Neurooncol.

[67] Levin VA, Bidaut L, Hou P, Kumar AJ, Wefel JS (2011). Randomized double-blind placebo-controlled trial of bevacizumab therapy for radiation necrosis of the central nervous system.. Int J Radiat Oncol Biol Phys.

[68] Pope WB, Lai A, Mehta R, Kim HJ, Qiao J (2011). Apparent diffusion coefficient histogram analysis stratifies progression-free survival in newly diagnosed bevacizumab-treated glioblastoma.. AJNR Am J Neuroradiol.

[69] Price TJ, Hardingham JE, Lee CK, Weickhardt A, Townsend AR (2011). Impact of KRAS and BRAF Gene Mutation Status on Outcomes From the Phase III AGITG MAX Trial of Capecitabine Alone or in Combination With Bevacizumab and Mitomycin in Advanced Colorectal Cancer.. J Clin Oncol.

[70] Varker KA, Biber JE, Kefauver C, Jensen R, Lehman A (2007). A randomized phase 2 trial of bevacizumab with or without daily low-dose interferon alfa-2b in metastatic malignant melanoma.. Ann Surg Oncol.

[71] Di Lorenzo G, Figg WD, Fossa SD, Mirone V, Autorino R (2008). Combination of bevacizumab and docetaxel in docetaxel-pretreated hormone-refractory prostate cancer: a phase 2 study.. Eur Urol.

[72] Friedman HS, Prados MD, Wen PY, Mikkelsen T, Schiff D (2009). Bevacizumab alone and in combination with irinotecan in recurrent glioblastoma.. J Clin Oncol.

[73] Hecht JR, Mitchell E, Chidiac T, Scroggin C, Hagenstad C (2009). A randomized phase IIIB trial of chemotherapy, bevacizumab, and panitumumab compared with chemotherapy and bevacizumab alone for metastatic colorectal cancer.. J Clin Oncol.

[74] Tol J, Koopman M, Cats A, Rodenburg CJ, Creemers GJ (2009). Chemotherapy, bevacizumab, and cetuximab in metastatic colorectal cancer.. N Engl J Med.

[75] Kreisl TN, Zhang W, Odia Y, Shih JH, Butman JA (2011). A phase II trial of single-agent bevacizumab in patients with recurrent anaplastic glioma.. Neuro Oncol.

[76] Vredenburgh JJ, Desjardins A, Reardon DA, Peters KB, Herndon JE (2011). The addition of bevacizumab to standard radiation therapy and temozolomide followed by bevacizumab, temozolomide, and irinotecan for newly diagnosed glioblastoma.. Clin Cancer Res.

[77] Blumenschein GR, Kabbinavar F, Menon H, Mok TS, Stephenson J (2011). A phase II, multicenter, open-label randomized study of motesanib or bevacizumab in combination with paclitaxel and carboplatin for advanced nonsquamous non-small-cell lung cancer.. Ann Oncol.

[78] Chang JW, Thongprasert S, Wright E, Tsang K, Kim HT (2011). An indirect comparison of bevacizumab plus cisplatin-gemcitabine and cisplatin plus pemetrexed treatment for patients with advanced first-line non-squamous non-small cell lung cancer in East Asia.. Asia Pac J Clin Oncol.

[79] Chang GC, Ahn MJ, Wright E, Kim HT, Kim JH (2011). Comparative effectiveness of bevacizumab plus cisplatin-based chemotherapy versus pemetrexed plus cisplatin treatment in East Asian non-squamous non-small cell lung cancer patients applying real-life outcomes.. Asia Pac J Clin Oncol.

[80] Picus J, Halabi S, Kelly WK, Vogelzang NJ, Whang YE (2011). A phase 2 study of estramustine, docetaxel, and bevacizumab in men with castrate-resistant prostate cancer: results from Cancer and Leukemia Group B Study 90006.. Cancer.

[81] McGonigle KF, Muntz HG, Vuky J, Paley PJ, Veljovich DS (2011). Combined weekly topotecan and biweekly bevacizumab in women with platinum-resistant ovarian, peritoneal, or fallopian tube cancer: results of a phase 2 study.. Cancer.

[82] Zhi J, Chen E, Major P, Burns I, Robinson B (2011). A multicenter, randomized, open-label study to assess the steady-state pharmacokinetics of bevacizumab given with either XELOX or FOLFOX-4 in patients with metastatic colorectal cancer.. Cancer Chemother Pharmacol.

[83] Moroney JW, Schlumbrecht MP, Helgason T, Coleman RL, Moulder S (2011). A phase I trial of liposomal doxorubicin, bevacizumab, and temsirolimus in patients with advanced gynecologic and breast malignancies.. Clin Cancer Res.

[84] Négrier S, Gravis G, Pérol D, Chevreau C, Delva R (2011). Temsirolimus and bevacizumab, or sunitinib, or interferon alfa and bevacizumab for patients with advanced renal cell carcinoma (TORAVA): a randomised phase 2 trial.. Lancet Oncol.

[85] Rastogi P, Buyse ME, Swain SM, Jacobs SA, Robidoux A (2011). Concurrent bevacizumab with a sequential regimen of doxorubicin and cyclophosphamide followed by docetaxel and capecitabine as neoadjuvant therapy for HER2- locally advanced breast cancer: a phase II trial of the NSABP Foundation Research Group.. Clin Breast Cancer.

[86] Yardley DA, Raefsky E, Castillo R, Lahiry A, Locicero R (2011). Phase II study of neoadjuvant weekly nab-paclitaxel and carboplatin, with bevacizumab and trastuzumab, as treatment for women with locally advanced HER2+ breast cancer.. Clin Breast Cancer.

[87] Sehgal R, Lembersky BC, Rajasenan KK, Crandall TL, Balaban EP (2011). A phase I/II study of capecitabine given on a week on/week off schedule combined with bevacizumab and oxaliplatin for patients with untreated advanced colorectal cancer.. Clin Colorectal Cancer.

[88] Wong NS, Fernando NH, Bendell JC, Morse MA, Blobe GC (2011). A phase II study of oxaliplatin, dose-intense capecitabine, and high-dose bevacizumab in the treatment of metastatic colorectal cancer.. Clin Colorectal Cancer.

[89] Grignol VP, Olencki T, Relekar K, Taylor C, Kibler A (2011). A phase 2 trial of bevacizumab and high-dose interferon alpha 2B in metastatic melanoma.. J Immunother.

[90] Jemal A, Siegel R, Ward E, Hao Y, Xu J (2008). Cancer statistics, 2008.. CA Cancer J Clin.

[91] Shih T, Lindley C (2006). Bevacizumab: an angiogenesis inhibitor for the treatment of solid malignancies.. Clin Ther.

[92] Scappaticci FA, Skillings JR, Holden SN, Gerber HP, Miller K (2007). Arterial thromboembolic events in patients with metastatic carcinoma treated with chemotherapy and bevacizumab.. J Natl Cancer Inst.

[93] Nalluri SR, Chu D, Keresztes R, Zhu X, Wu S (2008). Risk of venous thromboembolism with the angiogenesis inhibitor bevacizumab in cancer patients: a meta-analysis.. JAMA.

[94] Hapani S, Chu D, Wu S (2009). Risk of gastrointestinal perforation in patients with cancer treated with bevacizumab: a meta-analysis.. Lancet Oncol.

[95] Wu S, Kim C, Baer L, Zhu X (2010). Bevacizumab increases risk for severe proteinuria in cancer patients.. J Am Soc Nephrol.

[96] Ranpura V, Pulipati B, Chu D, Zhu X, Wu S (2010). Increased risk of high-grade hypertension with bevacizumab in cancer patients: a meta-analysis.. Am J Hypertens.

[97] Cortes J, Calvo V, Ramírez-Merino N, O'Shaughnessy J, Brufsky A (2011). Adverse events risk associated with bevacizumab addition to breast cancer chemotherapy: a meta-analysis.. Ann Oncol 2011 Oct 4.

[98] Clarke MJ, Stewart LA, Egger M, Smith GD, Altman DG (2001). Obtaining individual patient data from randomized controlled trials,. Systematic Reviews in Health Care.

