# Supplementary material for: Effect of Angiogenesis Inhibitor Bevacizumab on Survival in Patients with Cancer: A Meta-Analysis of the Published Literature
Source: PLoS One. 2012 Apr 23;7(4):e35629. doi: 10.1371/journal.pone.0035629 (PMC3335091; doi:10.1371/journal.pone.0035629)
Supplement: Table S1 — Characteristics of the trials included in this meta-analysis. (DOC) [file pone.0035629.s001.doc]

Table 1 Characteristics of the trials included in this meta-analysis

| Study | Location/Period | Trial Phase | Tumor Type | No. of Enrolled | No. of Analysis | Follow up (mo) | Commitment Therapy | BEV dose (mg/kg/wk) | Quality scores |
| --- | --- | --- | --- | --- | --- | --- | --- | --- | --- |
| Johnson (11) | USA | 2 | NSCLC | 99 | 98 | 14.7 | Paclitaxel andcarboplatin | 2.5 or 5 | 7 |
|  | - |  |  |  |  |  |  |  |
| Sandler (12) | USA | 3 | NSCLC | 878 | 867 | 19.0 | Paclitaxel and carboplatin | 5 | 8 |
|  | 7/2001-4/2004 |  |  |  |  |  |  |  |
| Herbst (13) | US | 2 | NSCLC | 122 | 81 | 15.8 | Docetaxel or pemetrexed | 5 | 7 |
|  | 10/2004-11/2005 |  |  |  |  |  |  |  |
| Reck (14) | Multiple countries 2/2005-8/2006 | 3 | NSCLC | 1043 | 986 | − | Cisplatin and gemcitabine | 2.5 or 5 | 7 |
| Brahmer (15) | US | 2/3 | NSCLC | 878 | 850 | − | Paclitaxel and carboplatin | 5 | 5 |
|  | - |  |  |  |  |  |  |  |
| Herbst (16) | Multiple countries | 3 | NSCLC | 636 | 636 | 19 | Erlotinib or cisplatin | 5 | 9 |
|  | 6/2005-4/2008 |  |  |  |  |  |  |  |
| Spigel (17) | US | 2 | SCLC | 215 | 215 | − | carboplatin plus etoposide | 5 | 8 |
|  | 3/2007-8/2008 |  |  |  |  |  |  |  |
| Hurwitz (18) | Multiple countries | 3 | Colorectal cancer | 813 | 790 | 18.0 | Irinotecan, bolus fluorouracil, and leucovorin | 2.5 | 8 |
|  | 9/2000-5/2002 |  |  |  |  |  |  |
|  |  |  |  |  |  |  |  |  |
| Kabbinavar (19) | Multiple countries 8/2000-7/2002 | 2 | Colorectal cancer | 209 | 204 | 14.8 | Bolus fluorouracil and leucovorin | 2.5 | 7 |
| Giantonio (20) | Multiple countries | 3 | Colorectal cancer | 829 | 572 | 28 | Oxaliplatin fluorouracil, and leucovorin | 5 | 7 |
|  | 11/2001-4/2003 |  |  |  |  |  |  |
|  |  |  |  |  |  |  |  |  |
| Saltz (21) | Multiple countries | 3 | Colorectal | 1401 | 1369 | 27.6 | Oxaliplatin, | 2.5 | 8 |
|  | 2/2004-2/2005 |  | cancer |  |  |  | fluorouracil, and eucovorin or capecitabine and oxaliplatin |  |  |
| Moehler (22) | Germany  2001-2006 | 2 | Colorectal cancer | 46 | 46 | 19.5 | capecitabine plus irinotecan | 7.5 | 6 |
| Tebbutt (23) | Multiple countries  7/2005-6/2007 | 3 | Colorectal cancer | 471 | 471 | 31 | capecitabine, and mitomycin | 2.5 | 7 |
| Stathopoulos (24) | Greece | 3 | Colorectal cancer | 222 | 222 | 36 | Leucovorin 5-fluorouracil and irinotecan | 2.5 | 7 |
|  | 9/2004-9/2008 |  |  |  |  |  |  |
|  |  |  |  |  |  |  |  |  |
| Kemeny(25) | US | 2 | Colorectal cancer | 73 | 73 | 30 | Fluorodeoxyuridine and dexamethasone | 2.5 | 7 |
|  | _ |  |  |  |  |  |  |
| Miller (26) | US | 3 | Breast cancer | 462 | 455 | 14.8 | Capecitabine | 5 | 7 |
|  | 11/2000-3/2002 |  |  |  |  |  |  |  |
| Miller (27) | US 12/2001-5/2004 | 3 | Breast cancer | 722 | 711 | 25.9 | Paclitaxel | 5 | 7 |
| Miles (28) | Multiple countries 3/2006-4/2007 | 3 | Breast cancer | 736 | 730 | 10.2 | Docetaxel | 2.5 or 5 | 9 |
| Brufsky (29) | Multiple countries 2/2006-6/2008 | 3 | Breast cancer | 684 | 684 | 15 | Taxane; gemcitabine; vinorelbine | 5 | 9 |
| Robert (30) | Multiple countries 12/2005-8/2007 | 3 | Breast cancer | 1277 | 1277 | 15.6 | capecitabine Taxane and anthracycline | 5 | 8 |
| Martin (31) | Multiple countries 12/2006-7/2008 | 2 | Breast cance | 282 | 191 | − | Paclitaxel | 5 | 8 |
| Yang (32) | USA 10/1998-9/2001 | 2 | Renal cell carcinoma | 116 | 116 | 27 | None | 2.5 or 5 | 8 |
| Escudier (33) | Multiple countries 6/2004-10/2005 | 3 | Renal cell carcinoma | 649 | 641 | 13.3 | Interferon-α | 5 | 9 |
| Rini (34) | Multiple countries 10/2003-7/2005 | 3 | Renal cell carcinoma | 732 | 715 | 24 | Interferon-α | 5 | 8 |
| Van Cutsem (35) | Multiple countries 7/2005-9/2006 | 3 | Pancreatic cancer | 607 | 583 | 6.7 | Gemcitabine and erlotinib | 2.5 | 7 |
| Kindler (36) | USA 6/2004-4/2006 | 3 | Pancreatic cancer | 602 | 540 | 11.3 | Gemcitabine | 5 | 9 |
| Burger (37) | Multiple countries 10/2005-6/2009 | 3 | Ovarian Cancer | 1873 | 1873 | 17.4 | Paclitaxel and carboplatin | 5 | 8 |
| Perren (38) | Multiple countries 12/2006-2/2009 | 3 | Ovarian Cancer | 1528 | 1528 | 19.4 | Paclitaxel and carboplatin | 2.5 | 9 |
| Kemeny (39) | US  − | 2 | Liver cancer | 56 | 56 | − | floxuridine and dexamethasone | 5 | 5 |
| Ohtsu (40) | Multiple countries 9/2007-12/2008 | 3 | Gastric Cancer | 774 | 774 | 11.4 | Cisplatin and gemcitabine | 2.5 | 10 |
