# Supplementary material for: Effect of Angiogenesis Inhibitor Bevacizumab on Survival in Patients with Cancer: A Meta-Analysis of the Published Literature
Source: PLoS One. 2012 Apr 23;7(4):e35629. doi: 10.1371/journal.pone.0035629 (PMC3335091; doi:10.1371/journal.pone.0035629)
Supplement: Table S2 — Subgroup analyses. (DOC) [file pone.0035629.s002.doc]

Table 2 Subgroup analyses

|  | Progression to free survival | | |  | Overall survival | | |  | One-year survival rate | | |
| --- | --- | --- | --- | --- | --- | --- | --- | --- | --- | --- | --- |
|  | n. of study | HR (95% CI) | p-value |  | n. of study | HR (95% CI) | p-value |  | n. of study | OR (95% CI) | p-value |
| Cancer type |  |  |  |  |  |  |  |  |  |  |  |
| Lung cancer | 7 | 0.68 | < 0.001 |  | 6 | 0.83 | < 0.001 |  | 5 | 1.20 | 0.063 |
|  |  | 0.62 to 0.73 |  |  |  | 0.75 to 0.92 |  |  |  | 0.99 to 1.45 |  |
| Colorectal cancer | 7 | 0.68 | < 0.001 |  | 8 | 0.81 | < 0.001 |  | 7 | 1.43 | < 0.001 |
|  |  | 0.59 to 0.78 |  |  |  | 0.75 to 0.89 |  |  |  | 1.23 to 1.65 |  |
| Breast cancer | 7 | 0.73 | < 0.001 |  | 6 | 0.94 | 0.606 |  | 6 | 1.25 | 0.007 |
|  |  | 0.67to 0.79 |  |  |  | 0.85 to 1.04 |  |  |  | 1.06 to 1.46 |  |
| Renal cell carcinoma | 3 | 0.63 | < 0.001 |  | 2 | 0.81 | 0.067 |  | 2 | 1.36 | 0.050 |
|  |  | 0.52 to 0.77 |  |  |  | 0.64 to 1.02 |  |  |  | 1.00 to 1.85 |  |
| Pancreatic cancer | 2 | 0.79 | < 0.001 |  | 2 | 0.97 | 0.606 |  | 2 | 1.13 | 0.348 |
|  |  | 0.70 to 0.89 |  |  |  | 0.85 to 1.10 |  |  |  | 0.88 to 1.45 |  |
| Ovarian cancer | 2 | 0.79 | 0.014 |  | 2 | 0.88 | 0.102 |  | 1 | 1.13 | 0.348 |
|  |  | 0.66 to 0.95 |  |  |  | 0.76 to 1.03 |  |  |  | 0.88 to 1.45 |  |
| Other cancers | 2 | 1.04 | 0.915 |  | 2 | 0.87 | 0.099 |  | 2 | 1.23 | 0.348 |
|  |  | 0.49 to 2.23 |  |  |  | 0.73 to 1.03 |  |  |  | 0.80 to 1.90 |  |
| Bevacizumab dose |  |  |  |  |  |  |  |  |  |  |  |
| Low | 12 | 0.73 | < 0.001 |  | 12 | 0.86 | < 0.001 |  | 11 | 1.42 | < 0.001 |
|  |  | 0.66 to 0.81 |  |  |  | 0.80 to 0.92 |  |  |  | 1.25 to 1.61 |  |
| High | 22 | 0.71 | < 0.001 |  | 19 | 0.88 | < 0.001 |  | 17 | 1.26 | < 0.001 |
|  |  | 0.67 to 0.75 |  |  |  | 0.83 to 0.93 |  |  |  | 1.14 to 1.39 |  |
| Chemotherapy regimen |  |  |  |  |  |  |  |  |  |  |  |
| Platinum or taxanes | 16 | 0.73 | < 0.001 |  | 14 | 0.85 | < 0.001 |  | 12 | 1.35 | < 0.001 |
|  |  | 0.68 to 0.78 |  |  |  | 0.80 to 0.90 |  |  |  | 1.21 to 1.52 |  |
| Nonplatinum or nontaxanes | 14 | 0.69 | < 0.001 |  | 14 | 0.89 | 0.002 |  | 12 | 1.25 | < 0.001 |
|  |  | 0.63 to 0.77 |  |  |  | 0.83 to 0.96 |  |  |  | 1.11 to 1.41 |  |
| Overall | 30 | 0.72 | < 0.001 |  | 28 | 0.87 | < 0.001 |  | 25 | 1.30 | < 0.001 |
|  |  | 0.68 to 0.76 |  |  |  | 0.83 to 0.91 |  |  |  | 1.20 to 1.41 |  |

HR = hazard ratio; CI = confidence interval; OR = odds ratio.
